# The Impact of Modified Two-Surgeon Technique for Laparoscopic Liver Resection on the Training of Surgeons-in-Training

**DOI:** 10.7759/cureus.38865

**Published:** 2023-05-11

**Authors:** Takahisa Fujikawa, Yusuke Uemoto, Taisuke Matsuoka

**Affiliations:** 1 Surgery, Kokura Memorial Hospital, Kitakyushu, JPN

**Keywords:** surgeons-in-training, on-the-job training, learning curve, modified two-surgeon technique, laparoscopic liver resection

## Abstract

Introduction

Although laparoscopic liver resection (LLR) has gained widespread acceptance over the last decade, it is associated with a much steeper learning curve than other laparoscopic procedures. We currently perform a modified two-surgeon technique for LLR. We assessed the effect of our LLR technique on the surgical outcome and the learning curve of surgeons-in-training when pure non-anatomical LLR was performed.

Methods

Between 2017 and 2021, 118 LLRs were conducted at our institution, 42 of which were pure non-anatomical LLRs performed by five surgeons-in-training (with a career of 6-13 years). The perioperative outcomes of these cases were compared to those performed by the board-certified attending surgeon. Regarding the learning curve of surgeons-in-training, the duration of operation was used as an index of the proficiency level, and the number of surgical cases in which the surgeons reached the median duration of operation was examined.

Results

Mortality was zero, and neither postoperative bleeding nor bile leak was experienced in the whole cohort. There were no differences between surgeons-in-training and the board-certified surgeon in the duration of the operation, intraoperative blood loss, rate of postoperative complications, or length of postoperative stay (LOS). Among the operations performed by five surgeons-in-training, the rate of LLR with a difficulty score of 4 or higher was 52% (30%-75%). Concerning the learning curve, all five surgeons-in-training gradually shortened the duration of operation for each additional case and reached the median duration (218 minutes) by experiencing a median of five cases (3-8 cases).

Conclusion

A modified two-surgeon technique during LLR is feasible, with a relatively low number of cases (five cases) required to shorten the duration of operation in non-anatomical LLR. This technique is safe and beneficial to the education of surgeons-in-training.

## Introduction

Since surgical techniques and management before and after surgery have improved, liver resection has become the most commonly performed operation for hepatic malignancy. Minimally invasive surgery has also been used successfully in liver surgery [1-4]. Regarding intraoperative hemorrhage and transfusion, laparoscopic liver resection (LLR) is thought to offer possible benefits over open surgery [1,5]. However, if bleeding occurs, LLR is more difficult to manage than the open approach, and LLR has a much steeper learning curve compared to other laparoscopic operations.

Blood loss, operative time, rate of conversion, complications, and length of postoperative stay (LOS) are outcomes related to the learning curve for LLR [6]. The learning curve in LLR has been shown to require 20 instances for minor LLR and 50 for major LLR, according to some articles in the literature [7,8].

We currently perform a modified “two-surgeon technique” for LLR [9,10]. Using a saline-linked ball-tipped electrocautery, the secondary surgeon concentrates on hemostasis, while the primary surgeon dissects liver parenchyma. This method enables quick and safe laparoscopic liver parenchymal transection that is comparable to open liver resection.

In the current paper, we assessed the effect of our LLR technique on the surgical outcome and the learning curve of surgeons-in-training when a pure minor (non-anatomical) LLR was performed.

## Materials and methods

Between 2017 and 2021, 118 LLRs were conducted at our institution, 42 of which were pure non-anatomical LLRs performed by five surgeons-in-training (with a career of 6-13 years). During the same period, eight minor LLRs were performed by the board-certified attending surgeon, which was regarded as the control group. Anatomical resection was defined as liver resection that was performed along the demarcation line after the Glissonean pedicle was occluded; liver resection of all other types (e.g., wedge resection) was regarded as non-anatomical resection.

Demographics, surgical treatments, and postoperative outcomes were obtained through a standardized review of the prospectively collected surgical database as well as hospital charts. Postoperative complications were assessed using the Clavien-Dindo classification (CDC) [11], and CDC class 2 or higher was considered relevant. Operative mortality was defined as death within 30 days of surgery.

To assess the difficulty level of LLR, the IWATE criteria, proposed at the Second International Consensus Conference on Laparoscopic Liver Resection, were used [12]. Briefly, the difficulty level is ranked on a scale of 0-12, and the difficulty index is further stratified into four groups: low (0-3), intermediate (4-6), advanced (7-9), and expert (10-12).

The perioperative outcomes of 42 LLRs performed by the surgeons-in-training were compared to those performed by the attending surgeon. Regarding the learning curve of surgeons-in-training, the duration of operation was used as an index of the proficiency level, and the number of surgical cases in which the surgeons reached the median duration of operation was examined. The Kokura Memorial Hospital Clinical Research Ethics Committee authorized the protocol of the current study (#21021002), which complied with the Declaration of Helsinki.

Surgical technique

**Figure 1:**
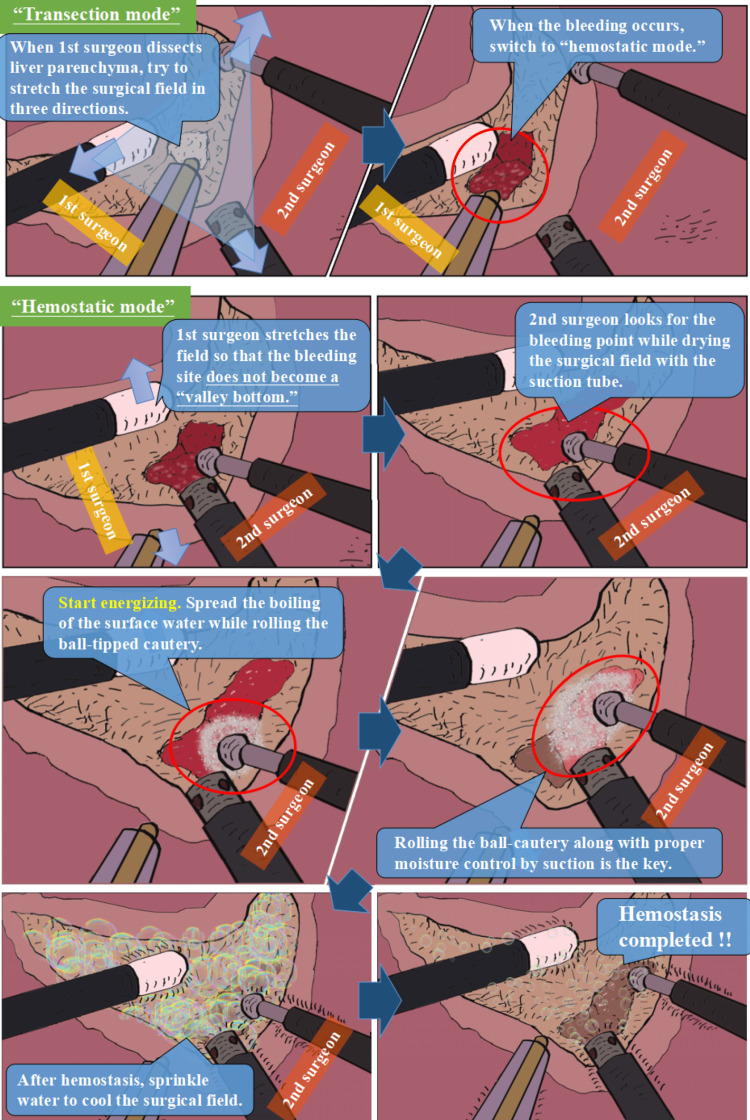
Outline of the modified two-surgeon technique in laparoscopic liver resection. Reproduced from [10] with permission

For LLR, we currently employ a modified two-surgeon technique [9,10]. The primary surgeon dissects the liver parenchyma, while the secondary surgeon uses a saline-linked ball-tipped electrocautery to achieve hemostasis. In our modified procedure, the “transection mode” and the “hemostatic mode” are maintained as separate sets to ensure adequate “role sharing,” and these modes are rigorously switched based on the surgical field situation (Figure 1).

An overview of the role sharing and mode switching in the modified two-surgeon technique during LLR is shown in Video 1. During the “transection mode,” the secondary surgeon can control minimal bleeding without altering modes. If bleeding occurs from a deep parenchymal fissure, the mode is changed to “hemostatic mode” promptly. The primary surgeon stretches the surgical field to help the secondary surgeon achieve hemostasis.

**Video 1:** Role sharing and mode switching in modified two-surgeon technique during laparoscopic liver resection (with narration). Reproduced from [10] with permission

Statistical analysis

While categorical data are reported as absolute numbers or percentages, continuous variables are shown as the median along with the interquartile range. Fisher’s exact probability test and the Mann-Whitney U-test were used, respectively, to compare categorical and continuous variables. The learning curve was assessed with linear regression analyses. The threshold for statistical significance was a two-sided P-value of 0.05. All statistical analyses were conducted using the graphical user interface for R (version 2.13.0, R Foundation for Statistical Computing, Vienna, Austria) known as EZR (Saitama Medical Center, Saitama, Japan) [13].

## Results

Among the 42 patients included in the study, no conversion to open hepatectomy was required. Mortality was zero, and neither postoperative bleeding nor bile leak was experienced in the whole cohort. There were no differences between the surgeons-in-training and board-certified surgeons in the duration of operation (218 versus 180 minutes), intraoperative blood loss (50 versus 25 mL), rate of postoperative complications (Clavien-Dindo class 2 or higher, 4.8% (one pneumonia and one congestive heart failure) versus 0%), and LOS (8 versus 9 days) (Table 1).

**Table 1:** Comparison of surgical outcomes between patients receiving laparoscopic liver resection performed by surgeons-in-training and by attending surgeons. Abbreviations: LLR: laparoscopic liver resection, RBC: red blood cell, CDC: Clavien-Dindo classification, CHF: congestive heart failure, LOS: length of postoperative stay

Variables	LLRs by surgeons in training (n = 42)	LLRs by attending surgeons (n = 8)	P-value
Difficulty index	4 (1-6)	4 (2-6)	0.890
Difficulty level by IWATE criteria	Intermediate	Intermediate	
Operative time (minutes)	218 (49-392)	180 (104-307)	0.346
Intraoperative RBC transfusion	1 (2.4%)	0 (0%)	1.000
Surgical blood loss (mL)	50 (5-560)	25 (5-60)	0.187
Postoperative complications (CDC ≥ 2)	2 (4.8%, pneumonia, CHF)	0	0.887
Operative mortality	0	0	-
LOS (days)	8 (3-17)	9 (7-14)	0.908

**Figure 2:**
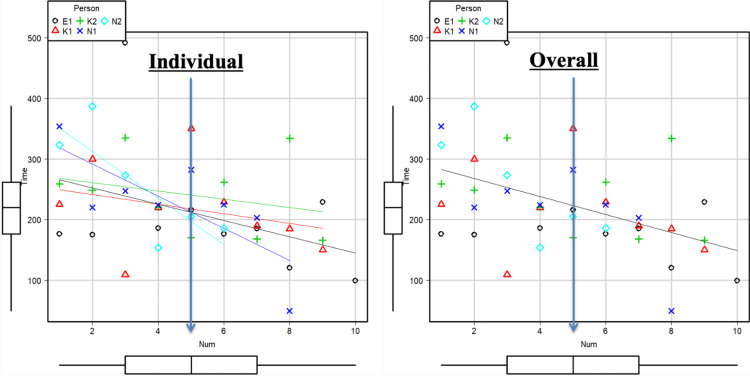
Scatterplots illustrating the correlation between the operative time and the number of LLRs performed by surgeons-in-training. Significant negative correlations were demonstrated between the operative time and the number of LLRs in either individual analyses (left graph) or overall analyses (right graph) (P = 0.002). Abbreviation: LLR: laparoscopic liver resection

Details of the operative characteristics and short-term outcomes of LLRs performed by each of the five surgeons-in-training are summarized in Table 2. Among the operations performed by five surgeons-in-training, the rate of LLR with difficulty index 4 or higher was 52% (30%-75%). Concerning the learning curve, all five surgeons-in-training gradually shortened the duration of operation for each additional case and reached the median duration (218 minutes) by experiencing a median of five cases (Figure 2). There was a statistically significant negative correlation between the operative time and the number of LLR performed by surgeons-in-training (P = 0.002). These negative correlations were demonstrated in either individual analyses or overall analyses.

**Table 2:** Details of operative characteristics and short-term outcomes of laparoscopic liver resections performed by each of the five surgeons-in-training. Abbreviations: DI: difficulty index, OT: operative time, CDC: Clavien-Dindo classification, LOS: length of postoperative stay

Surgeon	Number of patients	DI	Rate of DI ≥ 4	OT (minutes)	Number of cases to reach median OT	Operative blood loss (mL)	CDC ≥ 2	LOS (days)
#1 (E1)	10	3 (1-5)	55%	181 (99-392)	5	5 (5-320)	0	8 (3-11)
#2 (K1)	9	4 (1-5)	44%	200 (109-300)	5	50 (5-530)	0	9 (8-17)
#3 (K2)	9	3 (2-6)	30%	249 (166-335)	7	70 (5-410)	0	7 (4-9)
#4 (N1)	8	5 (1-6)	75%	225 (49-282)	5	28 (5-560)	1	9 (5-17)
#5 (N2)	6	5 (3-6)	67%	239 (154-387)	5	73 (5-260)	1	9 (8-16)
Total	42	4 (1-6)	52%	218 (49-392)	5	50 (5-560)	2 (4.8%)	8 (3-17)

## Discussion

In the current paper, we described the safety and efficiency of the modified two-surgeon technique during LLR for the education of surgeons-in-training. Using this strategy, a relatively low number of cases (five cases) was required to shorten the operative time in non-anatomical LLR. There was a significant negative correlation between the operative time and the number of LLRs performed by surgeons-in-training, and these negative correlations were demonstrated in either each individual case or the overall analysis.

Over the past decade, the incidence of LLRs has grown. LLR may offer advantages over open surgery in regard to intraoperative hemorrhage, intraoperative transfusion rate, and other short-term results [1,5]. In the Second International Consensus Conference on LLR [1], the use of a minor LLR was considered to be standard surgical practice. Nevertheless, the procedure is still in the assessment phase, and the major LLR is still in the exploration phase. LLR is associated with a significantly steeper learning curve than other laparoscopic operations. According to current reports in the literature, the LLR learning trajectory requires 20-40 cases for minor LLR and 40-60 cases for major LLR [7,8]. The current study demonstrates that the modified two-surgeon technique for non-anatomical LLR requires a relatively small number of cases (five cases) to reduce operative time.

The two-surgeon technique presented in the present article was initially used for open liver resections and was shown to improve a variety of outcomes [14-16]. We adapted this strategy to LLR [10]. This method enables swift laparoscopic liver parenchymal transection identical to that of open liver resection. Due to the clear division of roles in this method, rapid hemostasis is achievable. Standardization of a procedure for LLR, like the strategy in our surgical team, is vital for a learning curve. If operative methods or procedures vary depending on the senior surgeon, inexperienced surgeons will become perplexed. For the education of safe and confident LLRs, it is considered critical to train repeatedly and share procedures in a standardized manner.

**Figure 3:**
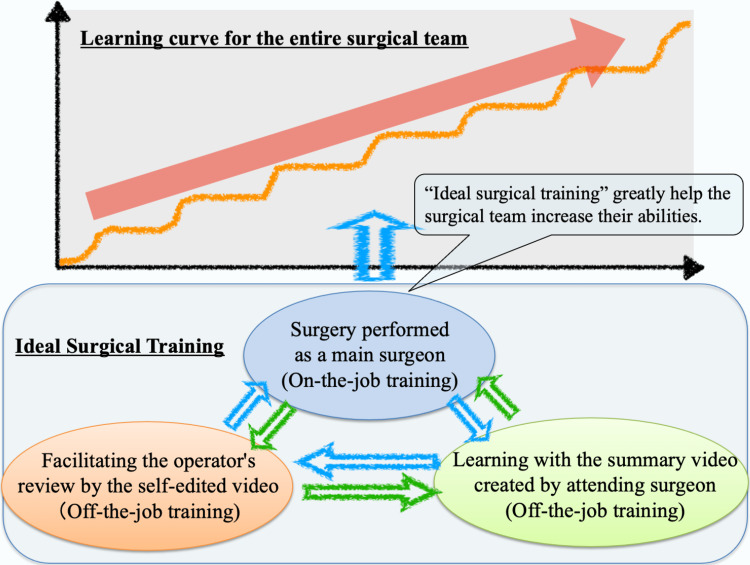
Concept of efficient surgical training using both on-the-job and off-the-job training and its positive effect on the learning curve of the entire surgical team. The efficient surgical training we have proposed consists of both off-the-job and on-the-job training, and it positively affects the learning curve of the entire surgical team.

Additionally, we believe that the modified two-surgeon technique in LLR is secure and beneficial not only for the training of inexperienced surgeons but also for improving the abilities of the surgical team as a whole. Figure 3 depicts our current surgical education system, in which both on-the-job and off-the-job training are coordinated effectively. Training inexperienced surgeons to recognize and discuss the concepts of “mode switching” and “role sharing” during LLR can enhance their LLR skills early in their careers. In addition, complete sharing of the tasks enables them to receive sufficient on-the-job training equivalent to open surgery. Consequently, the surgical team as a whole can enhance its capabilities. Even inexperienced young surgeons can learn the method quickly and contribute to the success of the surgical team at our facility. Despite the fact that LLR has a much steeper learning curve than other laparoscopic operations, the current method can help individual surgeons as well as the entire surgical team learn how to perform their duties more effectively.

There are some limitations to the current investigation. First, due to the retrospective nature of the study, it is of restricted use in determining the effect of this strategy on outcomes. Second, the sample size was insufficient; a larger sample size would likely result in more reliable recommendations. Lastly, a control group is required to demonstrate that our suggested approach is preferable to alternatives. Nonetheless, we are persuaded that this method is a precursor to the future standardization of LLR.

## Conclusions

The modified two-surgeon technique during LLR is safe and feasible for the education of surgeons-in-training. There was a significant negative correlation between the operative time and the number of LLR performed by surgeons-in-training. Using this strategy, a relatively low number of cases (five cases) is required to shorten the operative time in non-anatomical LLR.

## References

[1] Wakabayashi G, Cherqui D, Geller DA (2015). Recommendations for laparoscopic liver resection: a report from the second international consensus conference held in Morioka. Ann Surg.

[2] Nguyen KT, Laurent A, Dagher I (2009). Minimally invasive liver resection for metastatic colorectal cancer: a multi-institutional, international report of safety, feasibility, and early outcomes. Ann Surg.

[3] Belli G, Fantini C, D'Agostino A, Cioffi L, Langella S, Russolillo N, Belli A (2007). Laparoscopic versus open liver resection for hepatocellular carcinoma in patients with histologically proven cirrhosis: short- and middle-term results. Surg Endosc.

[4] Croner RS, Perrakis A, Hohenberger W, Brunner M (2016). Robotic liver surgery for minor hepatic resections: a comparison with laparoscopic and open standard procedures. Langenbecks Arch Surg.

[5] Zhang XL, Liu RF, Zhang D, Zhang YS, Wang T (2017). Laparoscopic versus open liver resection for colorectal liver metastases: a systematic review and meta-analysis of studies with propensity score-based analysis. Int J Surg.

[6] Nomi T, Fuks D, Kawaguchi Y, Mal F, Nakajima Y, Gayet B (2015). Learning curve for laparoscopic major hepatectomy. Br J Surg.

[7] Chua D, Syn N, Koh YX, Goh BK (2021). Learning curves in minimally invasive hepatectomy: systematic review and meta-regression analysis. Br J Surg.

[8] Fukumori D, Tschuor C, Penninga L, Hillingsø J, Svendsen LB, Larsen PN (2023). Learning curves in robot-assisted minimally invasive liver surgery at a high-volume center in Denmark: report of the first 100 patients and review of literature. Scand J Surg.

[9] Fujikawa T, Kawamoto H, Kawamura Y, Emoto N, Sakamoto Y, Tanaka A (2017). Impact of laparoscopic liver resection on bleeding complications in patients receiving antithrombotics. World J Gastrointest Endosc.

[10] Fujikawa T, Kajiwara M (2022). Modified two-surgeon technique for laparoscopic liver resection. Cureus.

[11] Dindo D, Demartines N, Clavien PA (2004). Classification of surgical complications: a new proposal with evaluation in a cohort of 6336 patients and results of a survey. Ann Surg.

[12] Wakabayashi G (2016). What has changed after the Morioka consensus conference 2014 on laparoscopic liver resection?. Hepatobiliary Surg Nutr.

[13] Kanda Y (2013). Investigation of the freely available easy-to-use software 'EZR' for medical statistics. Bone Marrow Transplant.

[14] Aloia TA, Zorzi D, Abdalla EK, Vauthey JN (2005). Two-surgeon technique for hepatic parenchymal transection of the noncirrhotic liver using saline-linked cautery and ultrasonic dissection. Ann Surg.

[15] Yamamoto Y, Ikai I, Kume M (1999). New simple technique for hepatic parenchymal resection using a Cavitron Ultrasonic Surgical Aspirator and bipolar cautery equipped with a channel for water dripping. World J Surg.

[16] Palavecino M, Kishi Y, Chun YS (2010). Two-surgeon technique of parenchymal transection contributes to reduced transfusion rate in patients undergoing major hepatectomy: analysis of 1,557 consecutive liver resections. Surgery.

